# Effects of intraspecific competition on the life cycle of the stonefly, *Nemurella pictetii *(Plecoptera: Nemouridae)

**DOI:** 10.1186/1472-6785-8-5

**Published:** 2008-04-16

**Authors:** Reimo Lieske, Peter Zwick

**Affiliations:** 1 Limnologische Fluss-Station des Max-Planck-Instituts für Limnologie, Schlitz, Germany

## Abstract

**Background:**

Considerable variation of life cycle duration in given insect species has been frequently recorded. Splitting of populations into cohorts with different life cycle lengths may occur, sometimes even between siblings from the same batch. Larval populations of the stonefly *Nemurella pictetii *in central Europe regularly split into a very fast developing and a normal univoltine cohort, leading to partial multivoltinism. The causes for such variation remain unknown but presumably act on the larval stage in which most of the life cycle is spent. We therefore studied possible effects of intraspecific competition on growth and development of larvae in the laboratory.

**Results:**

Intraspecific competition had important influence on growth and development of the larvae. High larval densities led to reduced growth and retarded development through interference, not through exploitative competition. All specimens were negatively affected by frequent encounters and the resulting disturbance. There were no dominant individuals able to grow and develop faster than the rest, at the expense of the others.

**Conclusion:**

Differences in life cycle length of *Nemurella pictetii *may result from different larval densities in different microhabitats and resultant different degrees of interference competition. Although competition alone probably does not cause splitting of populations into cohorts with different life cycle duration differences in size and development caused by other factors are certainly enhanced by intraspecific competition.

## Background

Flexible life cycle duration is widespread in insects [1,2], also among aquatic species [3]. Variation may concern an important portion of normal life cycle length. It arises mainly from differences in the duration of the larval stage in which most of the life cycle is spent [4]. The main causes for such differences are different temperatures, day length or food in the various habitats of the population (see, for example, the review by [5]).

Differences in life cycle length between individuals of a population at the same site have also repeatedly been reported for aquatic insects [6-10], but the causes are less easily explained. In the case of extended hatching from eggs, early hatching individuals are often assumed to have an advantage in development resulting from the combined effects of temperature and longer growth period [11-13]. Such explanations focus mainly on time as factor. However, what causes a population to split into several cohorts growing and developing at different speed? There are no satisfactory explanations so far. Often, cohort splitting occurs when larval density at the respective site is high (see below). Intraspecific competition may be important in this context as shown, for example, in tadpoles [14,15], water striders [16] and also stoneflies [17]. In the mentioned cases, some individuals grew better than the rest, at the expense of the remaining members of the cohort. Possible causes might be competition for space and access to resources (interference), or some direct competition for food (exploitative competition; after [18]).

To investigate possible causes of these frequently observed phenomena we used the stonefly, *Nemurella pictetii *Klapálek, 1900, as a model species to check for intraspecific competition as a possible cause of cohort splitting in experiments. Our experiments extended previous studies by testing competition under standardised laboratory conditions, for long periods of time. *N. pictetii *is particularly suitable for such study, for several reasons. In appropriate habitats like springs, seeps, and slow flowing spring runs *N. pictetii *may attain very high larval densities (see Table 1). The species is remarkable for a very flexible semi- to multivoltine life cycle (see references in [19]). It also exhibits cohort splitting, even between siblings from a single egg mass [20]. Some larvae hatching from eggs in spring attain the last instar within three months, emerging as adults and ovipositing in late summer of the same year. Other siblings from the same egg mass require a full year to complete development and emerge the next spring, together with offspring of their fast developing siblings [20,21]. Multimodal adult emergence described in other studies (for example, [22-24]) probably also reflects similar cohort splitting. At northern latitudes, *N. pictetii *may exhibit semivoltine life cycles [25].

**Table 1:** Larval densities of *Nemurella pictetii *per m^2 ^in different water bodies.

River	Density	Remarks	Publication	Publication
Spring Ravnkilde	Denmark	29292 (08)	Moss; first instar possibly not covered, n = 10	[42]
Broadstone Stream	England	1323 (09)	Substrates of all kind, n = 40	[55]
Tributary to stream Biała Przemsza	Poland	18365 (01)	Submerged macrophytes, larvae smaller than 1 mm not included, n = ?	[23]
Stream Vel'ký Javorník	Slovakia	ca. 900 (12)	Gravel and detritus, n = 2-3	[56]

Abbreviations: density = maximal larval density (in parentheses, the month when it occurs), n = number of parallel samples.

The present study attempts to cast light on these problems and is based on the following hypotheses: (1) intraspecific competition does influence growth and development of *N. pictetii*; (2) the type of competition involved is interference competition. To test these hypotheses, we first compared the immediate effect of different larval densities on speed of larval growth and development (1). In a second experiment we supplied excess food and indirectly estimated the amount of ingested food by determining the amount of faeces produced per unit time, under different larval densities (2). In doing so we assumed that speed of development is positively related to the amount of food ingested per unit time.

## Results

### Speed of growth and maturation experiment

**Figure 1:**
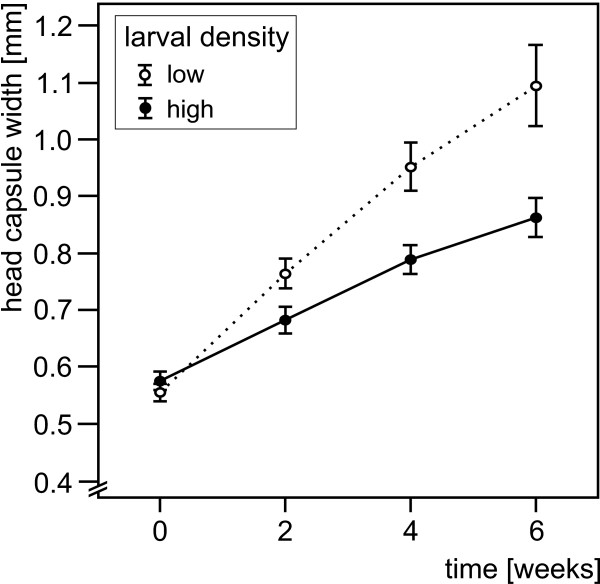
**Head capsule width of larvae of *Nemurella pictetii *(mean ± SD) with time during the speed of growth and maturation experiment**. Aggregate values are shown; low larval density (5 larvae per experimental unit): n = 10; high larval density (20 larvae per experimental unit): n = 9.

Larval density distinctly influenced both growth and development of the larvae. Larvae kept at low density grew faster and already after two weeks attained significantly larger HCWs than larvae kept at high density. The size differences between the two treatments increased further as the experiment continued (Table 2, Figure 1).

**Table 2:** Results of the repeated measures ANOVA of the head capsule width of larvae of *N. pictetii *in the speed of growth and maturation experiment.

Source of variation	d.f.	MQ	F	P
Larval density	1	0.364	61.988	< 0.001
Error (larval density)	17	0.006		
Time	3	2.010	1707.342	< 0.001
Larval density × time	3	0.092	78.010	< 0.001
Error (time)	51	0.001		

**Figure 2:**
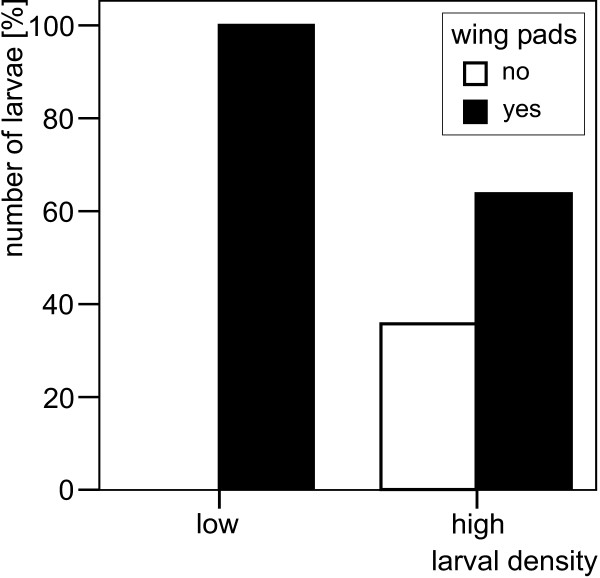
Percentage of larvae of *Nemurella pictetii *with and without wing pads, respectively, at the end of the speed of growth and maturation experiment.

Differences in development of larvae kept at the two different levels of density are evident from the degree of WPD (Mann-Whitney U-test, Z = -3.996, P < 0.001; Figure 2) While all larvae kept at low density possessed wing pads at the end of the experiment, the same was true of only 64% of the larvae kept at high density.

**Figure 3:**
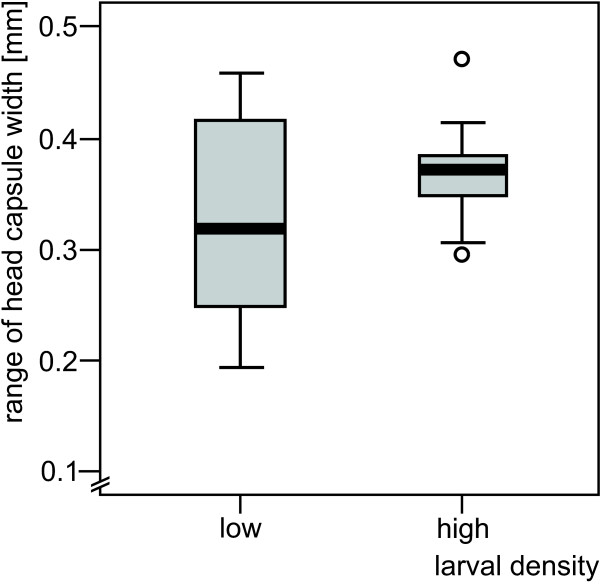
**Range of head capsule width of larvae of *Nemurella pictetii *per experimental unit at the end of the speed of growth and maturation experiment**. Low larval density (5 larvae per experimental unit): n = 10; high larval density (20 larvae per experimental unit): n = 9; in the boxplot, the box represents the interquartile range divided at the median; vertical lines indicate minimum and maximum values, circles identify outliers (i.e. 1.5–3 box lengths from the edges of the box).

In contrast, HCWR revealed no significant influence of larval density (Mann-Whitney U-test, Z = -0.816, P = 0.414). The range between the smallest and largest larva in each experimental unit was similar at the two larval densities (Figure 3).

SR was also not influenced by larval density (Mann-Whitney U-test, Z = -0.934, P = 0.350). At the end of the experiment, 94 and 93%, respectively, of the larvae survived at low and high density, respectively.

### Food ingestion experiment

**Figure 4:**
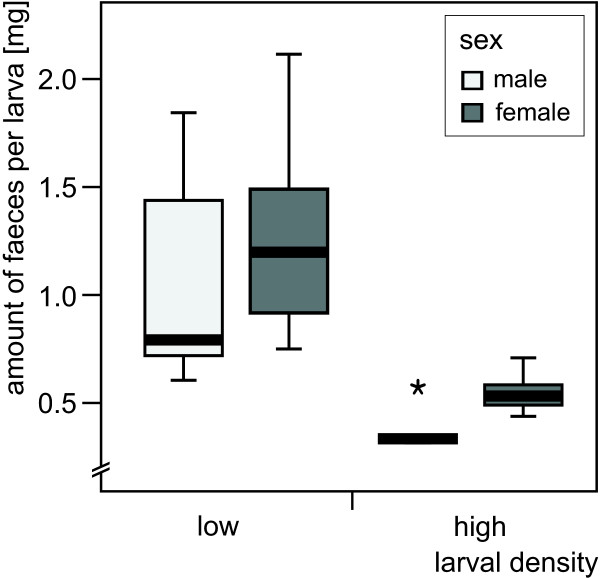
**Amount of faeces produced by individual *Nemurella pictetii *larvae (final three instars) during 66 hours when kept at low and at high larval density, respectively**. Low larval density (1 larva per experimental unit): n = 7 (males), n = 14 (females); high larval density (5 larvae per experimental unit): n = 5 (males), n = 9 (females); the star identifies an extreme value (more than 3 box lengths from the edges of the box), for further explanations of symbols see Figure 3.

Analysis of AF in the food ingestion experiment yielded significant differences among the four treatments. Larval density as well as sex distinctly influenced the amount of faeces produced by a larva (Table 3). Animals kept at high density produced fewer faeces than larvae kept at low density and females more than males. For example, male larvae kept at low density produced three times the amount of faeces produced at high density (Figure 4).

**Table 3:** Results of the ANOVA of the amount of faeces produced by larvae in the food ingestion experiment.

Source of variation	d.f.	MQ	F	P
Larval density	1	3.376	90.584	< 0.001
Sex	1	0.521	13.988	0.001
Larval density × sex	1	0.133	3.569	0.068
Error	31	0.037		

During the short term experiment, there was no food shortage. During the experiment, dry weight of the available biofilm decreased on average by only 8%, even at high larval density: from a mean DW of 49 mg (SD = 3) at the start to a mean of 45 mg DW (SD = 4) at the end of the experiment.

## Discussion

The results of the speed of growth and maturation experiment confirm our first hypothesis that intraspecific competition influences growth and development of *N. pictetii*. Both parameters were distinctly affected by larval density. High densities induced significantly slower growth and retarded development than observed at low densities. There were distinct (P < 0.001) differences in HCW as well as in WPD. However, intraspecific competition was not strong enough to distinctly influence survival rate (P = 0.350), which was over 90% at both factor levels.

During preliminary tests we had noticed a striking aggressiveness of larvae during encounters, at least at high densities. Larvae stood head to head, threatening with open mandibles. Actually, antennae but also cerci were often bitten off. Larvae behaved in ways similar to *Baetisr hodani *(Ephemeroptera) at high larval densities [26]. This author described larvae frequently pushed against others laterally, and conducted abrupt beats with the cerci against the competitor. Each larva may have a critical distance at which no others are tolerated, as described for other aquatic insect larvae with territorial behaviour (Plecoptera: [27], Odonata: [28]). For example, large nymphs of *Coenagrion resolutum *(Odonata) excluded smaller nymphs from areas with high food resources [28]. The favourable food supply might accelerate development of such larvae over others, and eventually lead to cohort splitting. However, analysis of HCWR suggests no similar mechanisms act in the present case. HCWR was only little wider at high than at low larval densities (P = 0.414). At the end of the experiment, there were no individuals in a unit that were strikingly larger than the rest; drastic suppression of some of the larvae by a few others appears improbable. The fact that HCWR of larvae was similar at both factor levels while HCW and WPD differed distinctly suggests two other causes.

First, *N. pictetii *larvae are very active and move around frequently [19,29,30]. At high larval density, disturbance during frequent encounters of larvae may retard development which would be interference competition. Stress during frequent encounters is supposed to reduce food ingestion and further increase locomotion. Hence, metabolic cost would increase at the expense of resources for growth. Our second consideration concerns exploitative competition. Shortage of high quality food (for example, densely microbially colonised leaf patches) may have caused retarded development at high larval density, affecting all individuals in a similar way.

The food ingestion experiment elucidated the kind of intraspecific competition more precisely. The distinctly negative correlation (P < 0.001) between larval density and amount of faeces produced by a larva does not suggest exploitative competition, for several reasons. On the one hand, biofilm is a high quality resource [31,32] that *N. pictetii *prefers [19]. On the other hand, enough biofilm remained during our short term experiments to exclude food shortage. Larvae used in the experiment were of comparable size and similarly far developed. Also, the biofilm used offered uniform food quality across the entire feeding plot. Therefore, interference (competition for space) seems to have been decisive, which supports our second hypothesis. Frequent encounters seem to have hindered ingestion of food. A recent study [30] found comparable evidence. Among other, the authors studied mass loss of conditioned alder leaves in relation to larval density of *N. pictetii *and also found a negative correlation. Like ourselves, they attributed their results to the high activity of the species and also regarded interference as cause of the correlation. In contrast, studies of intraspecific competition among slowly moving grazers like Trichoptera [33,34] or Gastropoda [35] regarded exploitative competition as more important factor than interference.

Although intraspecific competition importantly influences the life cycle length of *N. pictetii *it can hardly by itself cause the cohort splitting observed both in the field and in the laboratory. Egg development of *N. pictetii *is direct and synchronous, egg rests that are important in other stoneflies [36-38] do not occur in the present species ([20], own investigations). Instead, several factors may act in combination. Possibly, intraspecific competition resulting from high larval densities drives part of the population into suboptimal microhabitats with reduced availability of high quality food, i.e., benthic algae [19] where detritus or the like must instead be consumed. MACAN'S [39] reasoning to explain why the development of *Pyrrhosoma nymphula *(Odonata) takes 2 years in some microhabitats but 3 years in others is similar (compare [40,41]).

## Conclusion

In conclusion, intraspecific competition may importantly influence length of larval life and consequently the entire life cycle of the present species. Given the high larval densities of over 29000 specimens per square meter ([42]; see Table 1) observed in some habitats, interference competition must decisively influence the life cycle.

In our experiments, we observed interference competition, but no animal was able to profit at the expense of others. Therefore, cohort splitting observed in the field [20,21] cannot be explained by direct competition, provided the animals inhabit restricted areas with similar food quality. However, in more heterogeneous habitats displacement of some specimens to less favourable sites with lower food quality (e.g., detritus) can be conceived, especially in view of the aggressive behaviour observed in the present species. Size differences caused by different food qualities [19] may thus be enhanced through intraspecific competition by increasing territorial behaviour, as shown in water striders [16,43]. Further study is needed for a precise analysis of the situation.

## Methods

### Study organism

Larvae examined for speed of growth and development (experiment 1) came from the spring of the first order stream Breitenbach (50°40'N, 9°38'E, 280 m, see [44,45]). Size of specimens at the start of the experiment is given in Figure 1.

Larvae tested for food ingestion (experiment 2) were collected in the spring of the River Fulda (50°30'N, 09°56'E, 850 m, see [21]). The specimens were in the last three larval instars and were kept without food for 1 1/2 days before the experiment.

### Rearing condition

#### Rearing Systems and Experimental Units

The flowing water system used in the experiment on speed of growth and maturation consisted of a reservoir (25 l) from which an aquarium filter pump (Eheim, Typ 1026; 11.8 l min^-1^, Deizisau, Germany) pumped water into plastic tubes (∅ = 15 mm) with boreholes at regular distances. Each resulting water jet entered an experimental unit (poly-propylene, base area = 4.5 × 4.5 cm, volume = 70 ml) through a screen window (mesh size = 80 μm) in the lid. Water left via similar screen windows in the sides of the unit, flowing back to the reservoir *via *a trough. For details and illustrations, see [46] and [47].

A different flowing water system circulating a larger volume of water was used in the food ingestion experiment (for details see [19]). Each experimental unit consisted of two conical plastic beakers (poly-propylene, basal ∅ = 5.5 cm, volume = 170 ml) of which one had a gauze bottom (mesh size = 1500 μm) and was placed into the other beaker.

We used water from the Breitenbach in both experiments; for notes on its chemical properties see [48]. In the long lasting speed of growth and maturation experiment about half the water was replaced once a week. Random water samples revealed no change of water quality (conductivity, ammonium, nitrate and phosphate) during the experiments. Water temperature was 12°C, daylength was 16 h light:8 h dark in both experiments. Water temperature was recorded with a datalogger (1250 series, Grant Instruments [Cambridge] Ltd., Cambridgeshire, UK) at 10-min intervals.

#### Food

In the speed of growth and maturation experiment we used chips (area ≈ 11 cm^2^) from soft alder (*Alnus glutinosa*) shade leaves that had fallen in autumn. Air dried leaves were soaked in water for half a day, the chips punched with a metal tube and conditioned in aerated Breitenbach water for 2 weeks in the dark at 12 to 14°C before they were used as food. One leaf chip was randomly assigned to each experimental unit. Food was replaced twice a week.

In the food ingestion experiment we used unglazed clay tiles (5 × 5 × 0.5 cm; for details see [19]) after they had been exposed in trays in the Breitenbach for establishment of a natural biofilm [49]. This type of food resource has several advantages. It is the preferred food of *N. pictetii *[19], biofilm quantity and quality tend to be uniform across the entire area of a tile [50], and faeces fell directly through the gauze bottom into the lower collecting beaker when tiles were placed obliquely into the experimental units, with the biofilm on the lower side.

#### Experimental procedure and variables measured

The speed of growth and maturation experiment lasted for 44 days. Two factor levels (low and high larval density) were tested. Experiments started with 5 and 20 larvae, respectively, per experimental unit corresponding to 2500 and 9900, respectively, individuals/m^2 ^stream bottom. We used 10 and 9 experimental units, respectively, for experiments at low and high density, respectively. The increase in head capsule width across the eyes (HCW) during the experiment was recorded to document possible differences in growth and development between the treatments. HCW was measured four times: before the experiment, and then every second week using a dissecting microscope (WILD M5, Wild, Heerbrugg, Switzerland) combined with a digitizing tablet (Numonics 2200, Numonics, Montgomeryville, Pennsylvania, U.S.A). At the end of the experiment, we additionally recorded the range of HCWs in each experimental unit (HCWR), the number of individuals with wing pads as a measure of degree of maturation (WPD, wing pad development), and the survival rate (SR, percentage of larvae surviving since the start of the experiment). SR was calculated separately for each experimental unit, we here present the mean across all units. Since individual larvae could not be marked and identified, HCW and WPD were aggregated at the level of experimental units by calculating means per unit.

Two factor levels (low and high larval density, 1 and 5 larvae per unit, respectively, corresponding to 400 and 2100 individuals/m^2 ^stream bottom) were also tested in the food ingestion experiment. Lower numbers of larvae than in the previous experiment were used because the present larvae were much larger (mean HCW = 1.21 mm, SD = 0.09). We assessed the amount of ingested food indirectly by recording the amount of faeces produced (AF), on the assumption that larvae frequently disturbed by competitors would feed and defecate less. However, it was impossible to document the degree of disturbance precisely by counting the number of aggressive encounters between larvae during the experiment. The experiment lasted for 66 hours. Gut passage times in *N. pictetii *are only one to two hours [51], sufficiently many faecal pellets [52] were therefore produced during the experiment. *N. pictetii *grazes very efficiently on the biofilm, all detached pieces of biofilm are actually also eaten (personal observations). Therefore, only faeces dropped into the lower beaker of each experimental unit. A manual vacuum pump (MityvacII, Nalgene, Rochester, U.S.A.) was used to transfer the accumulated faeces to glass fibre microfilters (GF/C, ∅ = 25 mm, Whatman plc, Brentford, England). The filters were placed into small aluminium cups and dried at 105°C for 72 hrs (drying oven T6060, Heraeus, Hanau, Germany). Samples were allowed to cool in an exsiccator for three hours and were then weighed with an ultra microscale (4504 MP8, Sartorius, Göttingen, Germany). Before use, cups and filters had been in a furnace at 500°C for 2 hrs and were pre-weighed as described above. The amount of faeces produced during the experiment was the difference between the two readings.

Sexual dimorphism of *N. pictetii *is pronounced towards the end of the larval period [21,53]. Therefore, sex was included in the experimental design as an additional factor by running replicates separately for each sex. This as well as the use of only the last three larval instars was also intended to exclude that some individuals were much larger than the others, and might become dominant and possibly territorial. Shortly before molts, stonefly larvae do not feed (see references in [54]). To exclude any possible error caused by this we analysed only replicates in which no molts had occurred during the experiment. Seven experimental units with males and 14 with females were analysed at low density, and 5 and 9, respectively, at high density. Except for sex in the second experiment, larvae were randomly assigned to experimental units.

### Statistical analyses

We performed a repeated measures ANOVA to test for possible differences of HCW in the speed of growth and maturation experiment. Because variances within samples were not homogenous (Levene-Test), data were transformed (transformation exponent: -1). Differences in WPD, SR and HCWR were assessed with Mann-Whitney U-tests.

Possible differences in AF in the food ingestion experiment were also assessed with an ANOVA. Data were transformed (transformation exponent = -0.584) for the same reason as before.

## Abbreviations

AF: amount of faeces; HCW: head capsule width across the eyes; HCWR: range of head capsule width of larvae in single experimental units; SR: survival rate; WPD: wing pad development.

## Authors' contributions

The study was jointly planned by RL and PZ. RL performed the experiments and the statistical analyses. RL and PZ together wrote the present manuscript.
